# The bidirectional relationship between AMPK pathway activation and myokine secretion in skeletal muscle: How it affects energy metabolism

**DOI:** 10.3389/fphys.2022.1040809

**Published:** 2022-11-21

**Authors:** Mahdi Ahsan, Léa Garneau, Céline Aguer

**Affiliations:** ^1^ Department of Biochemistry, Microbiology and Immunology, University of Ottawa, Ottawa, ON, Canada; ^2^ Institut du Savoir Montfort –Recherche, Ottawa, ON, Canada; ^3^ Department of Physiology, Faculty of Medicine and Health Sciences, McGill University—Campus Outaouais, Gatineau, QC, Canada; ^4^ School of Human Kinetics, Faculty of Health Sciences, University of Ottawa, Ottawa, ON, Canada; ^5^ Interdisciplinary School of Health Sciences, Faculty of Health Sciences, University of Ottawa, Ottawa, ON, Canada

**Keywords:** myokines, AMPK, skeletal muscle, cell signalling, glucose metabolism, lipid metabolism

## Abstract

Myokines are peptides and proteins secreted by skeletal muscle cells, into the interstitium, or in the blood. Their regulation may be dependent or independent of muscle contraction to induce a variety of metabolic effects. Numerous myokines have been implicated in influencing energy metabolism *via* AMP-activated protein kinase (AMPK) signalling. As AMPK is centrally involved in glucose and lipid metabolism, it is important to understand how myokines influence its signalling, and *vice versa*. Such insight will better elucidate the mechanism of metabolic regulation during exercise and at rest. This review encompasses the latest research conducted on the relationship between AMPK signalling and myokines within skeletal muscles *via* autocrine or paracrine signalling.

## Introduction

The term myokine was coined by Pedersen et al., in 2003 to describe cytokines which are “produced and released by skeletal muscle” to affect “other organs” (Pedersen et al., 2003). At that time, the authors proposed interleukin 6 (IL-6) as a candidate to be classified as a myokine. Since then, the study of myokines has rapidly expanded to refine the definition of the term and apply it to several molecules including IL-6 (Pedersen and Febbraio, 2008; Pedersen, 2013; Garneau and Aguer, 2019). Our group has recently offered a clear definition for myokines as signalling peptides originating from skeletal muscle that are released in the muscle interstitium or in the blood to mediate functional or metabolic adaptations in distant and local tissues through autocrine, paracrine, or endocrine effects (Aguer et al., 2020). They may be regulated by muscle contraction and contribute to exercise-induced prophylactic and therapeutic metabolic effects (Krolner et al., 1983; Young, 1995; Praet and van Loon, 2007; Reimers et al., 2009; Aguer et al., 2020). However, myokines are also secreted at rest, independently of muscle contraction, as a result of basal cell signalling.

This definition makes it clear that myokines have a range of effects throughout the body. In fact, the influence of IL-6 on metabolism *via* central metabolic signalling molecule 5′ adenosine monophosphate-activated protein kinase (AMPK) has been suggested since the proposal of IL-6 as a myokine (MacDonald et al., 2003), setting the stage for the relationship between myokines and metabolism. It is thus unsurprising that many myokines have since been found to mediate exercise-induced metabolic effects through AMPK signalling.

AMPK is a critical signalling molecule central to glucose and lipid metabolism, protein synthesis, and autophagy (Yun and Zierath, 2006; Kjøbsted et al., 2018). Structurally, mammalian AMPK is a heterotrimeric complex composed of a catalytic α subunit, and regulatory β and γ subunits (Kim et al., 2016). The α and β subunits each have 2 isoforms (α1, α2, β1, and β2) while the γ subunit has 3 (γ1, γ2, and γ3). AMPK acts to maintain energy homeostasis by influencing glucose and lipid metabolism. As such, it may be activated by upstream effectors calmodulin-dependent protein kinase kinase (CaMKK), which is sensitive to intracellular calcium concentration, and liver kinase B1 (LKB1), which is a constitutively active kinase that activates AMPK when ATP levels are low. If either calcium or AMP content increases, the respective upstream kinase will phosphorylate AMPK at Thr172, activating its signalling pathway. As a central signalling molecule responsible for energy homeostasis, the phosphorylated form of AMPK (pAMPK) phosphorylates a plethora of proteins to elicit a multitude of downstream effects. It activates a variety of catabolic, energy producing pathways such as glucose and lipid metabolism, mitochondrial biogenesis, and autophagy. Simultaneously, it inhibits anabolic, energy consuming pathways such as macromolecule synthesis (Yun and Zierath, 2006; Hardie, 2015; Kim et al., 2016; Kjøbsted et al., 2018). It has been shown to impair glycogenesis by inhibitory phosphorylation of glycogen synthase while also promoting glycolysis by facilitating glucose uptake (Carling and Grahame Hardie, 1989; Wojtaszewski et al., 2002; Jørgensen et al., 2004; Hunter et al., 2011; Jeon, 2016).

This review aims to recapitulate the latest research conducted on the role of AMPK signalling in the metabolic function of several myokines and the discussion will contextualize the findings to exercise physiology and pathophysiology. To focus the scope of the paper on skeletal muscle, only the autocrine and paracrine effects are covered in the review.

## IL-6

As the most extensively researched myokine, IL-6 has long been associated with AMPK. Since it is a known mediator of energy homeostasis, the possibility of a relationship between AMPK activation and exercise-induced IL-6 release was first suggested in 2003 (MacDonald et al., 2003).

### IL-6-induced AMPK-mediated regulation of metabolism

MacDonald et al. reported a significant correlation between AMPK signalling and IL-6 in *vastus lateralis* of healthy young men during and after 60 min of exercise in low glucose conditions (MacDonald et al., 2003). This was further supported by *ex vivo* observations of 120 ng/ml IL-6 treatment increasing AMPK signalling for 60 min in rat *extensor digitorum longus* (EDL) (Kelly et al., 2004). In accordance, IL-6 knockout mice had lower levels of muscle AMPK phosphorylation at rest and in response to exercise compared to wild-type mice (Kelly et al., 2004; Ruderman et al., 2006). However, others have shown AMPK phosphorylation to be unaffected under similar conditions (O’Neill et al., 2013). Thus, it was suggested that IL-6 is only a minor regulator of AMPK phosphorylation in skeletal muscle.

Al-Khalili et al. determined IL-6 to specifically induce fatty acid oxidation *via* AMPK using an AMPKα siRNA, which reduced β-oxidation but did not affect glucose metabolism in human primary muscle cells (Al-Khalili et al., 2006). They showed that IL-6 increases GLUT4 expression, glucose uptake, glycogenesis, and glycolysis through phosphatidylinositol 3-kinase (PI3K) signalling while AMPK specifically mediates IL-6-induced lipid catabolism. The role of AMPK in IL-6-mediated β-oxidation was further confirmed by Carey et al. using dominant negative AMPK adenovirus-treated L6 myotubes, which failed to exhibit the increased palmitate oxidation seen in ad null cells (Carey et al., 2006). However, the authors also found the down regulation of AMPK in L6 myotubes reduced IL-6-mediated basal and insulin-dependent glucose uptake, suggesting the involvement of AMPK signalling in IL-6-induced glucose metabolism, contradicting the previous findings. Carey et al. also showed IL-6-induced AMPK activation in a dose-dependent manner to a level comparable to AICAR treatment lasting up to 120 min (Carey et al., 2006). 5-Aminoimidazole-4-carboxamide ribonucleotide (AICAR) is an AMP analog and a pharmacological activator of AMPK (Kim et al., 2016). The threshold for AMPK activation by IL-6 in L6 myotubes was determined to be 1 ng/ml and maximum activation was achieved between 10 and 100 ng/ml (Carey et al., 2006). Also, Glund et al. found muscle strips from the *vastus lateralis* of healthy male patients treated with 120 ng/ml IL-6 increased AMPK phosphorylation, which peaked at 15 min and returned to basal levels after 80 min (Glund et al., 2007). Further elucidating the mechanism of IL-6-induced AMPK activation, Kelly et al. reported that 120 ng/ml IL-6 increased the AMP:ATP ratio in rat EDL muscle *in vivo* which paralleled the increased AMPK activation (Kelly et al., 2009). Interestingly, inhibiting IL-6 induced AMPK activation with a β-adrenergic antagonist, propranolol, also inhibited IL-6-induced increase in cAMP and AMP:ATP levels. The association between IL-6, AMPK signalling, cAMP, and energy state were also observed in C2C12 myotubes and rat *gastrocnemius* muscle *in vivo* (Kelly et al., 2009). It was therefore suggested that IL-6 activates AMPK by increasing cAMP and AMP:ATP.

In studying the response to IL-6 treatment in the context of insulin resistance and type 2 diabetes, Jiang et al. found 120 ng/ml IL-6 similarly increased AMPK signalling in human primary muscle cells isolated from the *vastus lateralis* of healthy volunteers and individuals with type 2 diabetes (Jiang et al., 2013). Furthermore, Tang et al. recently reported that IL-6 treatment increased AMPK signalling and GLUT4 mRNA in palmitate-induced insulin resistant C2C12 myotubes. These effects were attenuated by silencing of IL-6, suggesting IL-6 may facilitate GLUT4-mediated glucose uptake irrespective of insulin sensitivity *via* AMPK signalling (Tang et al., 2019).

When considering plasma IL-6 concentrations in humans, the concentrations used in the above discussed studies appear supraphysiological. At rest, plasma concentration of IL-6 has been reported to be between 1.5 pg/ml and 6.4 pg/ml (Ostrowski et al., 1998b; Fernandez-Real et al., 2001; Garneau et al., 2020) and skeletal muscle interstitial concentration between 0 and 8 pg/ml, increasing between 641 and 2609 pg/ml post-exercise (Langberg et al., 2002; Rosendal et al., 2005). In athletes, 2.5 h of exercise has been shown to increase plasma IL-6 concentration 25-fold (∼35 pg/ml) (Ostrowski et al., 1998a). On the other hand, acute moderate-intensity exercise elevated plasma IL-6 to a lesser degree (∼3 pg/ml) in non-athletic healthy women (Garneau et al., 2020). In healthy males, low-intensity exercise was accompanied by a significant increase in interstitial IL-6 (∼1250 pg/ml) (Rosendal et al., 2005). So, although the principle of IL-6-induced dose-dependent increase in AMPK activation may hold true, it is difficult to directly relate most findings regarding pharmacological administration of IL-6 in a physiologically relevant context.

While much work has been done in studying IL-6, translating the current research *in vivo* in humans has proven difficult. Infusing recombinant IL-6 in healthy humans to reach circulating concentrations of ∼40 pg/ml did not result in the activation of AMPK in the *vastus lateralis* (Wolsk et al., 2010). However, this may be because the current study used a significantly lower concentration than that used in other *in vitro* studies, many of which did not investigate human cell lines (Kelly et al., 2004; Kelly et al., 2009; Carey et al., 2006; Glund et al., 2007). The authors noted the concentration used in their study was closer to that found in human plasma under physiological conditions (Pedersen and Febbraio, 2008). This suggests that physiological levels of IL-6 do not activate AMPK signalling in human skeletal muscle. Future research should aim to use more physiologically relevant concentrations of IL-6 to clarify the regulatory relationship between IL-6 and AMPK signalling when influenced by differing levels of exercise.

### AMPK-mediated regulation of IL-6

Activation of AMPK seems to negatively alter IL-6 mRNA expression and/or IL-6 secretion. Indeed, in 2008, Lihn et al. found AICAR-induced AMPK activation to suppress IL-6 mRNA expression and Il-6 secretion in a dose-dependent manner in human skeletal muscle cell line derived from the *rectus abdominis*, and in rat EDL *ex vivo* (Lihn et al., 2008). However, contrary to AICAR, treating EDL and soleus from wild-type mice with the allosteric AMPK activator, A-769662, induced a significant decrease in IL-6 secretion in the soleus but not in the EDL (Glund et al., 2009). This suggests that the AICAR-mediated decrease in IL-6 secretion was not directly due to increased AMPK activity, since AICAR is not a direct activator of AMPK while A-769662 is. Nonetheless, the fact that IL-6 secretion is inhibited by A-769662 in *soleus* strongly suggests that AMPK could regulate IL-6 secretion in some muscle types, at least in mice. Nylén et al. also confirmed AICAR-induced AMPK activation decreased IL-6 mRNA in primary cells from the *vastus lateralis* of healthy male volunteers as well as in L6 myotubes, and its secretion was reduced in the human primary muscle cells (Nylén et al., 2018). These effects were likely a result of transcriptional regulation as AICAR-induced AMPK signalling did not alter IL-6 mRNA half-life in L6 myotubes. However, even if A-769662, a direct AMPK activator, reduced IL-6 mRNA levels in human primary myotubes, it did not in L6 myotubes, suggesting that the effect of AICAR in L6 myotubes was not AMPK dependant. To confirm a causative relationship between AMPK and IL-6 signalling, it was demonstrated that siRNA-mediated knockdown of AMPKα1/2 in L6 myotubes caused an increase in IL-6 mRNA, which was slightly reduced by AICAR (Lihn et al., 2008). Furthermore, the reduction or absence of one of the AMPK subunits (α1, α2, or γ3) at the whole-body level in mice removed the inhibitory effect of AICAR on IL-6 mRNA expression and secretion, suggesting AMPK negatively regulates IL-6 expression in skeletal muscle (Glund et al., 2009). In accordance, Lantier et al. reported significantly elevated IL-6 expression in mouse *gastrocnemius* using a muscle specific AMPKα1α2 double-knockout model (Lantier et al., 2014). Muscle-specific AMPKα2 knockout mice with high fat diet (HFD)-induced obesity also demonstrated elevated IL-6 mRNA, serum, and interstitial protein concentrations (Chen et al., 2015). Taken together, the above discussed studies suggest that IL-6 expression may be regulated by the AMPK pathway through a negative feedback loop.

## IL-8

IL-8 has long been known to be an exercise-induced myokine, and it is believed to induce local angiogenesis (Nielsen and Pedersen, 2007). However, little research has been conducted on its relationship with AMPK.

### IL-8-induced AMPK-mediated regulation of metabolism

Gray & Kamolrat reported 20 pg/ml of IL-8 did not influence AMPK phosphorylation and insulin-stimulated glucose uptake in C2C12 muscle cells, whereas 1 ng/ml caused an increase in both (Gray and Kamolrat, 2011). The authors hypothesized that 1 ng/ml is similar to post-exercise interstitial levels and thus IL-8 increases glucose uptake by activating the AMPK pathway. Their hypothesis was based on literature on other myokines *in lieu* of research on post-exercise interstitial IL-8. To our knowledge, the post-exercise interstitial IL-8 level remains unknown. However, recent research has reported that at rest, plasma IL-8 ranges from approximately 3 pg/ml to 20 pg/ml while interstitial IL-8 has been reported just under 100 pg/ml (Kreiner et al., 2010; Garneau et al., 2020). A decrease in circulating levels of IL-8 in response to an acute bout of exercise has also been shown (Garneau et al., 2020). The 20 pg/ml used by Gray and Kamolrat is therefore reflective of plasma IL-8 levels at rest whereas 1 ng/ml is closer to, but still much higher than, that present in the interstitial fluid at rest. While it may be possible that interstitial IL-8 increases ten-fold in response to exercise, the lack of such data indicates that the concentration used by Gray and Kamolrat may be supraphysiological. Furthermore, the decrease in plasma IL-8 following exercise does not imply a similar response in muscle interstitial concentrations. If interstitial IL-8 does indeed increase post-exercise, it remains possible that IL-8 locally improves glucose uptake *via* AMPK. Further studies should therefore determine the effect of exercise on interstitial IL-8 concentrations and investigate this possibility.

### AMPK-mediated regulation of IL-8

Similar to IL-6, Lihn et al. reported AICAR-induced AMPK activation to be a potent dose dependent suppressor of IL-8 mRNA expression and secretion in human skeletal muscle cell line derived from the *rectus abdominis*, and in rat EDL *in vitro* (Lihn et al., 2008). In combination with the results of Gray & Kamolrat, the data suggest a possible regulatory feedback mechanism between AMPK and IL-8 which may be responsible for the lower plasma concentration of IL-8 post-exercise.

## IL-10

Interleukin-10 (IL-10) is an anti-inflammatory cytokine that is commonly secreted by immune cells to regulate the immune system, and extensive research has been conducted on its immunomodulatory role in various pathophysiological contexts (Couper et al., 2008; Sanjabi et al., 2009; Yao et al., 2013). However, studies have also investigated skeletal muscle derived IL-10 and its effects on various tissues (Hong et al., 2009; Dagdeviren et al., 2016).

### IL-10-induced AMPK-mediated regulation of metabolism

Although there have been numerous studies on IL-10, few have examined its relationship with AMPK. In 2014, Wang et al. found greater levels of pAMPK in the *tibialis anterior* of homozygous IL-10 deficient mice (Wang et al., 2014), suggesting IL-10 is a negative regulator of AMPK activation. Contrastingly, in rat skeletal muscle, IL-10 has been shown to increase in parallel with AMPK phosphorylation in response to exercise (Park et al., 2017). However, the measurements were not taken to analyze a causative relationship between the two. Furthermore, it is known that AMPK signalling is induced by exercise due to increased energy demands (Yun and Zierath, 2006; Kjøbsted et al., 2018). Further studies must be conducted to clarify the potential role of IL-10 in regulating AMPK activity. Future investigations must be cognisant of the recent findings of physiological plasma concentration of IL-10 in humans to be approximately 0.1 pg/ml (Garneau et al., 2020) as using excessively supraphysiological concentrations may jeopardize the biological relevance of the findings.

## IL-15

IL-15 was initially discovered as a T-cell growth factor and was later found to also be involved in neutrophil phagocytosis and macrophage differentiation (Burton et al., 1994; Grabstein et al., 1994; Ratthé and Girard, 2004; Zhen et al., 2014). It has since been found to be expressed in and secreted by muscle in response to exercise to mediate glucose and lipid metabolism (Nielsen et al., 2007; Quinn et al., 2009; Crane et al., 2015; Nadeau and Aguer, 2019).

### IL-15-induced AMPK-mediated regulation of metabolism

IL-15 treatment at high concentrations (1 ng/ml) for 2 h increased insulin-stimulated glucose uptake in C2C12 muscle cells (Gray and Kamolrat, 2011). This IL-15 effect was linked to an increased phosphorylation of AMPK. However, while the two were associated, a causative relationship was not established in that specific study. Conversely, using higher IL-15 concentrations (100 ng/ml) for 24 h, Krolopp et al. did not find IL-15 treatment to influence pAMPK levels despite an increase in glucose uptake (Krolopp et al., 2016). Therefore, it appears that either IL-15 can activate AMPK only in presence of insulin, or that the effects of IL-15 on AMPK phosphorylation are limited to short duration. This second hypothesis is probable due to IL-15’s limited half-life (30–60 min) (Bamford et al., 1996; Stoklasek et al., 2006; Han et al., 2011). In accordance, our group confirmed that an acute treatment of 3 h with concentrations of IL-15 above 10 pg/ml is associated with AMPK signalling in L6 myotubes, while longer treatment of 48 h needed very high IL-15 concentrations (≥10 ng/ml) to activate the AMPK pathway (Fujimoto et al., 2019). In any event, the findings of Krolopp et al. suggest that high IL-15 concentrations do not increase glucose uptake by activating AMPK, but rather that IL-15 activates glucose uptake through the Jak3/STAT3 signalling pathway. However, considering that muscle interstitial level of IL-15 has ben determined to range between 10 and 15 pg/ml (Pierce et al., 2015), and circulating concentrations of IL-15 at rest in women are around 2 pg/ml, elevating to around 3 pg/ml 24-h post-exercise (Garneau et al., 2020), this study used supraphysiological concentrations of IL-15 and may not represent IL-15 physiological function.

An increased AMPK pathway activation was also found in models of mice overexpressing IL-15 specifically in skeletal muscles. Indeed, we found that the *soleus* of muscle-specific-IL-15-overexpressing mice exhibited significantly greater ACC phosphorylation (pACC), but only a trend in the increase of pAMPK. The increase in pACC may suggest an increase in carnitine palmitoyl transferase 1 (CPT1) activity and thus an increased β-oxidation, but β-oxidation was not directly measured in our study. However, the EDL or *gastrocnemius* of IL-15 overexpressing mice did not exhibit increased AMPK signalling. This suggests the effect may be dependent on muscle fiber composition as the *soleus* is distinctly more oxidative than EDL and *gastrocnemius* muscles. This is possible as β-oxidation is generally more active in oxidative muscles due to a greater mitochondrial volume than glycolytic fibres (Howald et al., 1985; Ørtenblad et al., 2018). On the contrary, Fujimoto et al. found increased AMPK signalling in IL-15 overexpressing EDL and *soleus* but this increase only reached statistical significance in EDL (Fujimoto et al., 2019). These muscle-specific IL-15 overexpressing mice also showed an increased muscle GLUT4 translocation as well as an improvement in whole-body glucose tolerance. However, the study did not determine whether this improved glucose metabolism with IL-15 overexpression was related to the AMPK pathway.

To summarize, acute IL-15 treatment at high concentrations seems to increase AMPK activity in muscle cells, but it is still not clear whether in a physiological context, IL-15 acts through AMPK to improve muscle glucose and fatty acid metabolism.

### AMPK-mediated regulation of IL-15

Abbott et al. showed decreased IL-15 expression in skeletal muscle of AMPKα2 dominant negative mice and suggested AMPK activity to impact IL-15 expression (Abbott et al., 2012). This was further corroborated by Crane et al., who showed diminished IL-15 mRNA and protein levels in the *gastrocnemius* of skeletal muscle-specific AMPKβ1/2 deficient mice (Crane et al., 2015). *Soleus* and EDL muscles of these mice were not able to recover IL-15 mRNA upon AICAR treatment, whereas their wild-type counterparts demonstrated increased IL-15 mRNA. Furthermore, *in situ* electrical pulse stimulation of the sciatic nerve increased IL-15 mRNA in the *tibialis anterior* of wild-type but not of AMPK deficient mice, supporting the hypothesis that AMPK activation is necessary for contraction-induced IL-15 expression. Therefore, the current literature illustrates a one-way relationship between IL-15 and AMPK, where IL-15 is dependent on AMPK, but does not influence it in return under physiological conditions.

## IL-18

Interleukin-18 (IL-18) was discovered in 1989 and has since been found to be expressed in skeletal muscle, be increased in plasma following exercise, to increase fatty acid oxidation in isolated mouse *soleus* muscle, and to reduce insulin resistance in mice (Nakamura et al., 1989; Plomgaard et al., 2005; Lindegaard et al., 2013; Garneau et al., 2020).

### IL-18-induced AMPK-mediated regulation of metabolism

Although extensive research has deemed it critical in metabolic homeostasis, research on the association between IL-18 and AMPK is limited (Lindegaard et al., 2018). Lindegaard et al. demonstrated IL-18 to be an activator of skeletal muscle AMPK *in vitro* and *in vivo* (Lindegaard et al., 2013). Treatment with 1 ng/ml and 10 ng/ml recombinant IL-18 for 30 min induced AMPK phosphorylation in L6 muscle cells. Isolated mouse *soleus* muscle strips demonstrated increased AMPK phosphorylation and palmitate oxidation in response to 100 ng/ml recombinant IL-18. These results were confirmed in an *in vivo* model showing that overexpression of IL-18 in mouse *tibialis anterior* resulted in elevated AMPK signaling and CPT1 expression, suggesting an increased β-oxidation. The role of IL-18 signalling in mediating AMPK activity was confirmed in an IL-18 receptor deficient mouse model that showed impaired AMPK signalling and increased muscle triglyceride content. These data suggest IL-18 mediates muscle fatty acid oxidation *via* the AMPK pathway. However, the concentration of IL-18 used in these *in vitro* experiments were far greater than the ∼300 pg/ml present in circulation under physiological conditions (Garneau et al., 2020). Similarly, *in vivo* IL-18 overexpressing muscle exhibited up to 40-times greater IL-18 content than the wild-type muscle (Lindegaard et al., 2013) while IL-18 is only increased by approximately two-fold in response to exercise (Garneau et al., 2020). Thus, future research should investigate whether this relationship is consistent in humans using physiologically relevant concentrations.

## ANGPTL4

Angiopoietin-like protein 4 (ANGPTL4) was named by the HUGO Gene Nomenclature Committee shortly after its discovery in 2000 due to its similarity to other members of the angiopoietin family (Zhu et al., 2012). Since then, exercise-induced increases in skeletal muscle ANGPTL4 gene expression and plasma concentrations have been found, along with implications in exercise-mediated lipid metabolism (Catoire et al., 2014).

### ANGPTL4-induced AMPK-mediated regulation of metabolism

Recently, Chang et al. suggested ANGPTL4 as a regulator of exercise-induced AMPK phosphorylation through a possible negative feedback mechanism (Chang et al., 2018). Treatment with 45, 450, and 4500 ng/ml ANGPTL4 was found to increase AMPK phosphorylation in C2C12 myotubes, with 45 ng/ml being the most effective. A time-course study found this response to be active 1 min after treatment and waning after 60 min. ANGPTL4 mRNA and protein expression, and AMPK activity were also increased in *soleus* and *gastrocnemius* of exercised mice. The co-regulatory relationship was confirmed by an ANGPTL4 knockout mouse model, which exhibited impairments in exercise-induced AMPK phosphorylation in skeletal muscle. Interestingly, pACC levels were higher in the skeletal muscle of non-exercising ANGPTL4 knockout mice than their wild-type counterparts, suggesting differential regulation based on the metabolic condition. It is important to note that some of the concentrations used in this study were supraphysiological as physiological plasma ANGPTL4 has been reported to range between 4 and 100 ng/ml (Kersten et al., 2009; Catoire et al., 2014; Sabaratnam et al., 2018). It is interesting that the ANGPTL4 concentration closest to physiological exhibited the best response and higher concentrations did not elicit greater AMPK signalling, as this may suggest the response is saturated beyond a certain threshold. Future studies investigating the signalling relationship between ANGPTL4 and AMPK should be designed with these findings in mind and aim to use more biologically relevant concentrations of ANGPTL4.

### AMPK-mediated regulation of ANGPTL4

Research suggests that the activation of AMPK inhibits ANGPTL4 expression in skeletal muscle. Indeed, transcript and protein levels of ANGPTL4 in C2C12 myotubes were swiftly and significantly reduced in response to AICAR and metformin, which activates AMPK by impairing ATP production to increase AMP:ATP (Owen et al., 2000; Catoire et al., 2014; Kim et al., 2016; Rena et al., 2017). In accordance with these findings, treatment with AMPK inhibitor Compound C and a knockdown of AMPKα1/2 both countered the suppressive effect of AMPK on ANGPTL4 mRNA expression in human myotubes. The effect of AMPK in inhibiting ANGPTL4 expression was confirmed *in vivo* in mouse *vastus lateralis* since overexpressing AMPK reduced ANGPLT4 expression, while dominant-negative AMPK mutants showed increased ANGPTL4 expression.

Furthermore, Catoire et al. reported that in both C2C12 cells and human primary myotubes, oleic acid increased the mRNA expression of ANGPTL4 whereas AMPK activation decreased ANGPTL4 mRNA and protein in C2C12 cells, mouse *gastrocnemius*, and human primary myotubes. They reported ANGPLT4 to be regulated by exercise in two ways. First, following one-legged exercise experiments in humans, they reported ANGPTL4 mRNA and protein concentrations to be greater in the non-exercising muscles—likely due to increased exercise-induced plasma fatty acid concentrations. Second, plasma ANGPTL4 was significantly increased in response to acute exercise (3 h) but not 2 weeks of intense endurance training. Furthermore, using transgenic mice which overexpress ANGPTL4 in skeletal muscle, they determined the upregulation of this myokine impairs skeletal muscle fatty acid uptake. In combination with ANGPTL4’s known role of inhibiting lipoprotein lipase (LPL) activity (Yoshida et al., 2002), the authors proposed that the FFA-induced increase in ANGPTL4 expression inhibited lipoprotein lipase (LPL) activity in non-exercising muscle to prevent lipid overload, whereas AMPK inhibits this to promote fatty acid oxidation. Thus, the authors suggested that exercise-induced AMPK-mediated downregulation of ANGPTL4 facilitates β-oxidation by reducing the impairment of LPL activity to empower VLDL-derived triglyceride metabolism (Catoire et al., 2014).

Taken together, the literature shows a bi-directional relationship between ANGPTL4 and AMPK, where ANGPTL4 increases AMPK signalling which in turn downregulates it. Some have therefore suggested the two are involved in a negative feedback mechanism (Chang et al., 2018).

## BDNF

Brain-derived neurotrophic factor (BDNF) was suggested to be a contraction-induced myokine in experimentation with L6 myotubes (Matthews et al., 2009).

### BDNF-induced AMPK-mediated regulation of metabolism

Matthews et al. treated L6 myotubes with 1, 10, and 100 ng/ml of BDNF and reported a dose-dependent increase in AMPK phosphorylation, which was linked to an increase in fatty acid β-oxidation (Matthews et al., 2009). The role of AMPK in mediating BDNF-induced fatty acid catabolism was verified by treatments with Compound C and dominant negative AMPK adenovirus, where both inhibited BDNF-induced β-oxidation. These *in vitro* data on myotubes were confirmed in muscle tissue as BDNF treatment increased AMPK phosphorylation and subsequent fatty acid oxidation in rat EDL *ex vivo*. Yang et al. also reported exposure to 100 ng/ml BDNF to increase AMPK phosphorylation in C2C12 cells (Yang et al., 2019). Verifying this, muscle-specific BDNF knockout mice demonstrated decreased AMPK signalling in the *gastrocnemius* while fasted. Consequently, fatty acid oxidation and fasting-induced AMPK-mediated autophagy signalling were also diminished. These data suggest BDNF is involved in β-oxidation and autophagy *via* AMPK phosphorylation in skeletal muscle.

The lowest concentrations used by Matthews et al. (1 and 10 ng/ml) are more similar to those found in humans under physiological conditions than the concentrations utilized by Yang et al.. Indeed, Máderová et al. reported serum BDNF concentrations of ∼35 ng/ml in young participants and ∼20 ng/ml in elderly participants, while plasma concentration was ∼6 ng/ml for both. The authors reported exercise to increase circulating BDNF concentrations in the elderly participants, while active young participants inherently demonstrated elevated circulating BDNF. These results, in conjunction with the Matthews and Yang papers, may guide future studies on the role of exercise-induced BDNF in AMPK signalling and muscle metabolism.

## CCL5

Chemokine ligand 5 (CCL5) was recently identified as a contraction-reducible myokine in C2C12 myotubes (Ishiuchi et al., 2018). Electrical pulse stimulation decreased CCL5 concentration and secretion from C2C12 myotubes.

### CCL5-induced AMPK-mediated regulation of metabolism

AICAR-induced AMPK activation was also found to decrease CCL5 secretion, suggesting the effect of exercise on the level of CCL5 in muscle may be mediated by AMPK. However, AICAR treatment increased *Ccl5* gene expression, suggesting AMPK-mediated post-translational regulation of this myokine (Ishiuchi et al., 2018). Unfortunately, there is sparse research on CCL5 as a myokine and further research should be conducted to identify physiological concentrations present in humans and to further elucidate its role in skeletal muscle.

## FGF21

Fibroblast growth factor 21 (FGF21) was first identified as a hepatokine and later discovered to also be an exercise-induced myokine (Nishimura et al., 2000; Tanimura et al., 2016). The physiological circulating concentration of FGF21 has been found to be around 200 pg/ml (Garneau et al., 2020) and evidence of AMPK-mediated effects of FGF21 has been demonstrated in various studies.

### FGF21-induced AMPK-mediated regulation of metabolism

Knockdown of FGF21 in C2C12 myoblasts decreased AMPK mRNA abundance (Liu et al., 2017). In addition, FGF21 deficiency was found to significantly impair AMPK phosphorylation in the skeletal muscle of HFD-induced obese mice, which was linked to increased inflammation (Kim et al., 2019). This finding was corroborated in a study investigating the anti-diabetic mechanism of ampelopsin, a bioactive component of *Ampelopsis grossedentata*. Zhou et al. found the improvements in insulin sensitivity by ampelopsin was dependant of increased FGF21 expression due to the activation of the AMPK pathway (Zhou et al., 2016). Indeed, where ampelopsin treatment exhibited an increase in FGF21 and pAMPK, FGF21 knockdown inhibited ampelopsin-induced AMPK phosphorylation in palmitate-treated L6 myotubes, suggesting AMPK activation from this drug is dependent on FGF21. Furthermore, upon suggesting a pathway for AMPK-mediated regulation of FGF21, Vandanmagsar et al. implicated FGF21 contribution to skeletal muscle mass, energy expenditure, insulin signalling, and glucose utilization in skeletal muscle of mice with an altered muscle fatty acid oxidation due to muscle-specific *Cpt1b* deletion (Vandanmagsar et al., 2016).

### AMPK-mediated regulation of FGF21

As briefly mentioned in the previous section, the role of AMPK in the regulation of FGF21 expression has been demonstrated by Vandanmagsar et al. *via* a mouse model possessing a muscle-specific deletion of *Cpt1b* (Vandanmagsar et al., 2016). While Cpt1b^−/−^ mice demonstrated elevated AMPK activation (Wicks et al., 2015), primary myotubes derived from Cpt1b^−/−^ mice also showed elevated FGF21 mRNA and protein which was normalized upon exposure to the AMPK inhibitor Compound C (Vandanmagsar et al., 2016). Exposure to the Akt inhibitor A-674563 yielded similar results as Compound C, showing that both AMPK and Akt were involved in increased FGF21 levels. The authors were able to mimic the FGF21 increase in human primary myotubes by combined exposure to fatty acid and pharmacological inhibition of CPT1b by etomoxir. Treatment with Compound C and A-674563 once again exhibited similar results as with Cpt1b^−/−^ mice. Thus, they suggested that the increase in FGF21 occurs *via* an AMPK-mTOR-Akt axis. Similarly, muscle-specific AMPKα2 knockout mice with HFD-induced obesity also demonstrated elevated FGF21 mRNA (Chen et al., 2015).

### FGF21-induced AMPK-mediated regulation of other functions

FGF21 also induces the expression of myogenic genes *via* the AMPK pathway, as made apparent by the reduction of these effects following the inhibition of AMPK (Liu et al., 2017). Upon increasing FGF21 expression in C2C12 cells and treatment with either an AMPK activator or an AMPK inhibitor, Liu et al. found FGF21 induced AMPK phosphorylation to promote myogenesis. It was also noted that AMPK activation increased myosin heavy chain-I levels. Seeing as the levels of sirtuin 1 (SIRT1) and peroxisome proliferator-activated receptor gamma coactivator 1-alpha (PGC1α) also increased, they suggested the effect occurs through the SIRT1-AMPK-PGC1α pathway. PCG1α is a major modulator of energy metabolism and SIRT1 is a deacetylase which has been implicated in its regulation (Liang and Ward, 2006; Cantó and Auwerx, 2009). Furthermore, incubating C2C12 cells with mouse recombinant FGF21 exhibited a dose-dependent increase in pAMPK, while Compound C treatment attenuated the inhibitory downstream effects of FGF21 on TNFα-induced muscle atrophy (Kim et al., 2019). The role of the FGF21-AMPK pathway in the regulation of inflammation was confirmed as FGF21-treated myotubes demonstrated decreased inflammation and the effect was absent when AMPK was inhibited by Compound C (Kim et al., 2019).

Together, these data suggest a central role of FGF21 in the multiple AMPK-mediated pathways involving glucose metabolism, lipid metabolism, and myogenesis.

## FSTL-1

Follistatin-like 1 (FSTL-1) was first identified from a mouse osteoblastic cell line as it was found to be regulated by transforming growth factor β-1 (TGFβ1), and named TGFβ-stimulated clone 36 (TSC-36) (Shibanuma et al., 1993). The authors noted the similarity of its amino acid sequence with active-binding protein follistatin. Scientists later adopted the name FSTL-1 and found it to be a proinflammatory molecule (Miyamae et al., 2006). It has since been reported to be expressed in and secreted by cardiomyocytes (Oshima et al., 2008) and human primary skeletal muscle cells (Görgens et al., 2013). Circulating concentrations of FSTL-1 has been found to range from 1.06–18.49 ng/ml at rest (Widera et al., 2009; Görgens et al., 2013). Serum concentrations increased to 20.1 ± 3 ng/ml immediately after acute exercise, and normalized to basal concentration by 120 min post-exercise (Görgens et al., 2013).

### FSTL-1-induced AMPK-mediated regulation of metabolism

A study has found FSTL-1 induced time-dependent (200 ng/ml FSTL-1 for up to 360 min) and dose-dependent (0.02, 0.06, 0.2, 0.6, and 1.8 μg/ml FSTL-1 for 1 h) increase in AMPK signalling in L6 myotubes (Lee et al., 2017). A similar time-course study on primary differentiated rodent myoblasts verified these results. The authors reported that FSTL-1 induced AMPK-mediated glucose uptake as treatment with 200 ng/ml FSTL-1 for 3 h increased myotube glucose uptake, while Compound C and AMPK siRNA negated the effect. Furthermore, this was suggested to occur *via* the CAMKK pathway of AMPK activation as FSTL-1 increased intracellular calcium and treatment with STO-609, a CAMKK-specific inhibitor, suppressed AMPK phosphorylation and subsequent glucose uptake.

Altogether, this illustrates the role of FSTL-1 in increasing AMPK signalling to induce glucose uptake in skeletal muscle cells. However, much remains to be known about this relationship. Most pertinently, the interstitial concentrations of FSTL-1, to our knowledge, has not yet been reported. This will allow future studies to better tailor their methods to ensure biological relevance. Future studies may also investigate whether FSTL-1 also induce AMPK signalling *via* the AMP:ATP pathway, and if AMPK is involved in regulating it in return.

## LIF

Leukemia inhibitory factor (LIF) was first purified and cloned in 1987 (Gearing et al., 1987). At the time, it was known for its involvement in macrophage differentiation and proliferation. Since then, it has been found to be induced by exercise in skeletal muscle cells and associated with AMPK, with basal circulating concentration of LIF in healthy physically fit participants ranging between 26 and 29 pg/ml (Broholm et al., 2008).

### LIF-induced AMPK-mediated regulation of metabolism

Nylén et al. recently reported AICAR-induced AMPK activation to decrease LIF mRNA in human primary skeletal muscle cells (Nylén et al., 2018). When assayed alongside AMPK activators A-769662 and phenformin, AICAR was curiously the only one which reduced LIF mRNA in both human primary cells and L6 myotubes. AICAR-induced AMPK activation did not influence LIF mRNA stability in human or rat muscle cells. Seeing as the direct AMPK activator, A-769662, did not have the same effect as AICAR, it is possible that AICAR’s influence on LIF may not be through AMPK. However, knockdown of AMPKα1/2 in L6 myotubes increased LIF mRNA, supporting a role of AMPK signalling in the transcriptional regulation of LIF in skeletal muscles. Future research should aim to clarify how exactly AMPK influences LIF.

## METRNL

Metorin-like protein (METRNL) was initially discovered as a neurotrophic factor and an adipokine (Jørgensen et al., 2012; Li et al., 2014). Shortly thereafter, it was reported as an exercise-induced myokine which is present in circulation of healthy humans at concentrations of 993–1,313 pg/ml (Rao et al., 2014; Chung et al., 2018). Only a few studies have investigated associations between METRNL and AMPK in skeletal muscle to elucidate the molecular mechanisms of METRNL as a myokine.

### METRNL-induced AMPK-mediated regulation of metabolism

In 2018, Jung et al. demonstrated that treatment of C2C12 myotubes with up to 200 μg/ml of METRNL increased pAMPK in a dose-dependent manner, and intravenous administration of 2 µg of METRNL per day for 8 weeks lead to improved insulin sensitivity and counteracted high-fat-diet-induced suppression of AMPK phosphorylation in mice (Jung et al., 2018). Where METRNL reduced palmitate-induced nuclear translocation of immune regulating transcription factor nuclear factor kappa B (NFκB), phosphorylation of NFκB inhibitor IκBα, and palmitate-induced insulin resistance, knockdown of AMPK attenuated these effects of METRNL treatments. AMPK knockdown also suppressed the effects of METRNL on PGC1α expression, establishing AMPK is a mediator for numerous METRNL-induced cellular effects.

Lee et al. exposed C2C12 myotubes to electrical pulse stimulation for up to 36 h and confirmed a time-dependent increase in METRNL expression and secretion in relation with AMPK phosphorylation (Lee et al., 2020). METRNL seems to be involved in AMPK signalling in response to contraction since the effect of electrical pulse stimulation on AMPK phosphorylation was decreased upon METRNL knockdown. A parallel increase in AMPK and METRNL was also observed in the *quadriceps femoris* of mice following chronic exercise (1 h per day for 3 weeks). Furthermore, METRNL treatment increased AMPK phosphorylation and glucose uptake in time-dependent (100 ng/ml for up to 180 min) and dose-dependent (up to 300 ng/ml for 60 min) manners in C2C12 myotubes. This increased glucose uptake in response to METRNL was dependent on AMPK since Compound C and AMPKα2 knockdown abrogated this effect. The involvement of a muscle METRNL-AMPK pathway improving glucose homeostasis was confirmed in healthy, obese, and diabetic mouse models (Lee et al., 2020). The authors also demonstrated that METRNL exhibits anti-diabetic effects *via* calcium-mediated AMPK signalling in C2C12 myotubes and mouse primary myoblasts. However, the exact mechanism by which the myokine increases intracellular calcium remains open for future research endeavours. Furthermore, the concentrations of METRNL used in that specific study are magnitudes above physiological circulating concentrations. Current literature thus demonstrates high concentrations of METRNL to regulate blood glucose through AMPK signalling. Future research may also focus on identifying the interstitial concentrations of METRNL in response to exercise as this may provide better physiological context to the findings of Lee et al.

## SPARC

Secreted protein acidic and rich in cysteine (SPARC) was identified as an exercise-induced myokine capable of inhibiting colon tumorigenesis almost a decade ago (Aoi et al., 2013) and has longer been known to associate with AMPK to influence GLUT4 expression (Song et al., 2010).

### SPARC-induced AMPK-mediated regulation of metabolism

SPARC and AMPK were found together in an anti-SPARC immunoprecipitation assay performed on COS-7 primate kidney cells overexpressing flag-tagged SPARC and myc-tagged AMPKα1, demonstrating their physical association. Confocal microscopy of COS-7 cells also showed the two to be colocalized. *In vivo* interaction was suggested by co-immunoprecipitation of the endogenous proteins in HepG2 human hepatic cells and confirmed in rat skeletal muscle. Accompanying the physical interaction, SPARC has been suggested to influence AMPK phosphorylation in L6 myotubes (Song et al., 2010). Overexpressing SPARC increased both AMPK phosphorylation and GLUT4 expression, and SPARC inhibition by siRNA reduced AICAR-induced AMPK activation and subsequent GLUT4 expression (Song et al., 2010).

In studying the effects of whole-body SPARC-knockout in mice, an Ingenuity Canonical Pathway Analysis (ICPA) placed AMPK signalling in the top 10 of 396 inhibited pathways (Aoi et al., 2019). Accordingly, whole-body SPARC-knockout mice demonstrated lower AMPK phosphorylation in skeletal muscle after running when compared to wild-type mice, and acute SPARC treatment induced AMPK phosphorylation in *gastrocnemius* of the SPARC-knockout mice. ICPA also identified AMPKγ3 in the top 10 of 564 upstream regulators of SPARC gene expression. The authors reported SPARC to induce glucose uptake in EDL, but not in AMPKγ3-knockout mice. It was noted that SPARC-induced AMPK signalling and glucose uptake was more pronounced in EDL than in the *soleus*. This may be due to the EDL being more glycolytic compared to the more oxidative *soleus*.

Aoi et al. estimated SPARC may be responsible for 10–20% of total exercise-induced AMPK pathway activation in skeletal muscle (Aoi et al., 2019). SPARC-induced AMPK activation was dose-dependent in C2C12 myotubes treated with 0.2, 1, and 2 μg/ml of SPARC, while treatment with 1 μg/ml of SPARC for 10 min in human skeletal muscle cells activated AMPK and promoted glucose uptake. The concentration of SPARC used in the experiments (1 μg/ml) was similar to the estimated basal interstitial concentration (2.4 μg/ml) in mice, suggesting that SPARC may also be implicated in the mechanism of AMPK phosphorylation under non-exercising physiological conditions. However, Garneau et al. reported a more modest physiological circulating SPARC concentration of approximately 200 ng/ml in humans at rest (Garneau et al., 2020). Nonetheless, it is possible that interstitial levels of SPARC are higher in humans, but it remains unknown at this time. Future research should therefore aim to determine interstitial levels of SPARC in humans and investigate whether these observations translate to a human model at physiologically relevant concentrations.

### AMPK-mediated regulation of SPARC

AICAR-induced AMPK activation enhanced SPARC expression in a dose- and time-dependent manner in L6 myoblasts (Song et al., 2010). The nature of AMPK’s influence on SPARC was further supported by the decrease in SPARC mRNA and protein expression upon knockdown of AMPKα1 in L6 cells.

Taken together, the research indicated SPARC and AMPK to influence each other. SPARC increases AMPK phosphorylation which in turn increases SPARC expression. As such, further research should be conducted to elucidate the regulatory nature of this relationship. More work must also be done to determine the mechanism of AMPK activation resulting from increased SPARC expression.

## Apelin

Apelin was first identified as a novel myokine by Besse-Patin et al., in 2014, where they reported exercise-induced increases of apelin expression and secretion in human primary muscle cells (Besse-Patin et al., 2014). Prior to its identification as a myokine, Dray et al. found apelin to phosphorylate endothelial nitric oxide synthase (eNOS) and developed eNOS deficient mice for further study (Dray et al., 2008).

### Apelin-induced AMPK-mediated regulation of metabolism

In *in vitro* studies using muscle cells from the *soleus* of eNOS deficient mice, apelin was found to increase AMPK phosphorylation in time-dependent (1.55 μg/ml for 10–60 min) and dose-dependent (1.55 pg/ml to 15.5 ng/ml for 20 min) manners (Dray et al., 2008). As confirmed by mice lacking AMPK activity in muscle, and by eNOS deficient mice, apelin enhances glucose uptake *via* AMPK in *soleus* muscle, an effect that was completely inhibited when treated with Compound C. Acute treatment (15–60 min) with 1.55 ng/ml of apelin also induced time-dependent AMPK pathway activation in human myotubes from young and aged donors, confirming the effect in human muscle cells (Vinel et al., 2018). Together, this provides evidence for a role of AMPK in apelin-induced muscle glucose uptake *in vitro* and *in vivo*. However, it is important to note that the concentrations used in the above studies are much greater than the ∼40 pg/ml that has been reported in circulation of healthy humans (Chen et al., 2021). During their dose-response study, Dray et al. reported the concentration nearest to this (1.55 pg/ml) did not elicit a significant response but 155 pg/ml did. This suggests that the observations may be biologically relevant, but it is difficult to contextualize at this time because the physiological interstitial concentration of apelin is unknown.

### AMPK-mediated regulation of other functions

Using a dominant negative AMPK mouse model, Vinel et al. demonstrated the necessity of AMPK in apelin-induced myogenesis and protein synthesis (Vinel et al., 2018). Chronic treatment (4 days) with 1.55 ng/ml apelin increased grip strength and cross-sectional area of the *tibialis* muscle in wild-type aged mice but failed to do so in mice lacking AMPK activity. Based on these results, the authors suggested apelin supplementation to hold potential in revitalizing muscle in aging *via* myogenesis and muscle hypertrophy.

## Irisin

Irisin was first discovered as the secreted product of cleaved FNDC5, a driver of white adipose tissue browning, and implicated in exercise-induced thermogenesis independent of age and fitness level (Boström et al., 2012; Huh et al., 2014).

### Irisin-induced AMPK-mediated regulation of metabolism

Irisin was found to cause a dose-dependent (6, 20, 62, and 190 ng/ml) phosphorylation of AMPK in L6 cells, with 62 ng/ml eliciting maximum response, which increased glucose uptake, as confirmed by an AMPKα2 knockdown (Lee et al., 2015). This is an interesting observation as circulating concentration of irisin in humans is reported to be ∼3.5–4.5 ng/ml (Jedrychowski et al., 2015). While it may be possible that the signalling response observed by Lee et al. was overemphasized by the supraphysiological concentrations used, the highest concentration failing to elicit the greatest response may also suggest a threshold beyond which the response is saturated. Nonetheless, the authors provided further insight into irisin-mediated signalling by using the antioxidant N-acetylcysteine (NAC) to suggest reactive oxygen species (ROS) generation to be the mechanism of irisin-induced AMPK activation, as NAC treatment reduced AMPK phosphorylation and subsequent glucose uptake (Lee et al., 2015). Treatment with 62 ng/ml irisin for 1 h increased intracellular Ca^2+^ while inhibition of CaMKK prevented AMPK phosphorylation. Interestingly, NAC did not inhibit intramyocellular Ca^2+^ increase whereas the calcium-inhibitor BAPTA prevented ROS generation. This suggests the involvement of calcium signalling prior to ROS in activating AMPK, but AMPK signalling was not measured in response to BAPTA. Thus, it remains inconclusive if calcium and ROS are sequentially involved in irisin-induced AMPK phosphorylation. Together, the research indicates irisin to be involved in increasing glucose uptake through Ca^2+^ and ROS-mediated AMPK pathways. This also implicates irisin in exercise-induced glucose uptake as calcium and ROS are both increased in the muscle during contraction.

Irisin’s effect on AMPK phosphorylation has also been demonstrated in insulin resistant C2C12 myotubes cultured in high-glucose/high-fat medium, and in *gastrocnemius* of mice with diet-induced type 2 diabetes (Xin et al., 2016). Xin et al. not only suggested AMPK as a mediator of irisin-induced muscle glucose uptake, as previous studies have done, but also lipid metabolism, as AMPKα2 knockdown inhibited irisin-induced β-oxidation in insulin resistant C2C12 myotubes.

Recent research has suggested that irisin promotes insulin sensitivity through AMPK signalling, protects against palmitate-induced insulin resistance, and prevents high glucose-induced cytotoxicity (Yano et al., 2020). Overexpressing irisin in C2C12 muscle cells resulted in the phosphorylation of insulin receptor β (IRβ) comparable to control cells treated with insulin. Furthermore, irisin overexpressing cells were protected from palmitate-induced insulin resistance. Compound C significantly diminished pIRβ levels in irisin-overexpressing cells, suggesting irisin to induce AMPK-mediated IRβ phosphorylation. Furthermore, irisin overexpressing C2C12 cells cultured in a high glucose environment demonstrated noticeably lower viability in the presence of Compound C. In the same environment, irisin overexpressing cells with shRNA-mediated knockdown of insulin receptor were not viable, implicating insulin receptor activation to be a necessary component for the protection provided by irisin. Yano et al. also corroborated the effects of irisin on AMPK and muscle glucose uptake *in vivo*. *Soleus* muscle from mice injected with 20 ng/ml of irisin for 4 weeks also possessed greater GLUT4 and glycogen content (Yano et al., 2020). Thus, irisin plays an interconnected role between exercise, lipid, and glucose metabolism through AMPK signalling, suggesting a potential therapeutic role in the context of diabetes.

## Myonectin

In 2012, the term myonectin was inadvertently assigned to two different proteins. Lim et al. named C1q Tumor Necrosis Factor α-related protein isoform 5 (CTRP5) as myonectin while Seldin et al. did so with CTRP15 (Lim et al., 2012; Seldin et al., 2012). Since then, the literature shows discrepancies in which is officially considered myonectin (Seldin and Wong, 2012; Otaka et al., 2018; Garneau and Aguer, 2019; Lee and Jun 2019).

### Myonectin-induced AMPK-mediated regulation of metabolism

Meanwhile, some research exists on the relationship between AMPK and CTRP5, but a direct link between AMPK and CTRP15 is yet to be found (Seldin et al., 2012; Otaka et al., 2017) Moreover, it has yet to be demonstrated whether CTRP5 possesses the characteristics of a myokine. On the other hand, CTRP15 has been shown to be predominantly expressed in mouse skeletal muscle, and Seldin et al. noted that CTRP15 expression was greater in the *soleus* than in the *plantaris* of mice. Furthermore, they observed increased myonectin expression in C2C12 myotubes compared to myoblasts (Seldin et al., 2012). While this does not confirm whether CTRP15 is secreted by the skeletal muscle, and thereby meets the definition of a myokine, it provides a stronger basis for CTRP15 being a myokine than CTRP5. Though we provide here information on both CTRP5 and CTRP15 due to the inconsistency in the literature, Seldin et al. have made a compelling case for CTRP15 as the myokine myonectin. Many authors and manufacturers have chosen to follow suite, and as such, we concur with the identification of myonectin as CTRP15 (Seldin et al., 2012; Seldin and Wong, 2012; Otaka et al., 2017; Lee and Jun 2019; Aviscera BioScience, 2021; Creative Diagnostics, 2021; My BioSource, 2021.). Circulating concentration of CTRP15 in humans has been shown to be ∼50 μg/ml (Li et al., 2018). Further research should be conducted to investigate if CTRP15 interacts with AMPK. Conversely, further insight is also required to clarify whether AMPK influences CTRP15 expression or secretion in return.

## Myostatin

Discovered in 1997, myostatin was later found to be a negative regulator of muscle growth (McPherron et al., 1997; McPherron and Lee, 2002).

### Myostatin-induced AMPK-mediated regulation of metabolism

Myostatin has since been found to regulate glucose metabolism *via* AMPK in skeletal muscles (Chen et al., 2010). The association was first demonstrated through myostatin-treated C2C12 myotubes (0.375, 0.75, 1.5, and 3 μg/ml for 6 h and 1.5 μg/ml for 0.5, 1, 6, and 12 h) showing a dose- and time-dependent AMPK phosphorylation. Myostatin phosphorylates both AMPKα at Thr172 and AMPKβ at Ser108. This was confirmed in fasted myostatin knockout mice, which showed downregulation of AMPK activation. Chen et al. suggested the involvement of both the AMP:ATP and Ca^2+^ pathway in AMPK phosphorylation following myostatin treatment. This was based on the observations that ATP levels were reduced in myostatin-treated HeLa cells, yet LKB1-deficient HeLa cells sustained dose-dependent AMPK signalling, and CAMKKβ-deficient C2C12 myotubes exhibited reduced pAMPK levels. The involvement of AMPK in myostatin-induced glycolysis was confirmed as Compound C inhibited lactate production while the overall glucose consumption, as determined by glucose uptake, was unaffected in C2C12 myotubes. These findings suggest that myostatin-induced glycolysis in muscle is dependent on AMPK activation.

Although there seems to be a consensus in the literature for the role of myostatin in glucose metabolism *via* the AMPK pathway, contradictory findings on the nature of that role requires clarification. Indeed, Kazemi reported that resistance-exercise decreased plasma myostatin in humans (Kazemi, 2016), concurring with findings in mice EDL which exhibited increased AMPK activation when myostatin was inactivated (Zhang et al., 2011). Furthermore, the studies reviewed here used markedly higher concentrations than what is found in circulation under physiological conditions. Levels of circulating myostatin have been reported to be 9.08–9.12 ng/ml in healthy young men (Kazemi, 2016), ∼45 ng/ml in a cohort of healthy middle-aged men and women (Wang et al., 2012), and 59.01 ± 17.36 ng/ml in elderly men (Shabkhiz et al., 2020). Future studies should therefore attempt to clarify the nature of the relationship between myostatin and AMPK signalling.

## Pros and cons of research models

The studies included in this paper make use of tissue culture, rodent models, and human models. While the latter is the best reflection of myokine signalling in humans, the former two are not without their advantages. Two-dimensional tissue culture has proven to be an efficient method of study for many decades. As several studies in this paper utilize pharmacological treatments, 2D cell culture is an effective model as it allows for the even distribution of the treatment (Duval et al., 2017). However, recent research has found that the composition of commonly used media is not physiologically representative. McKee and Komarova reported that Dulbecco’s modified Eagles medium, a commonly used medium in muscle cell studies, contains excessive concentrations of sodium, calcium, chloride, bicarbonate, sulfate, and glucose compared to human plasma (McKee and Komarova, 2017). The authors make a fair point to argue that the increased electrolyte and glucose availability may confound current results and altering the media composition to better reflect the physiological environment may yield more representative data. Furthermore, there has been a recent push to make use of three-dimensional cell culture models to better represent *in vivo* conditions (Takahashi et al., 2018). While 2D models may be advantageous for the distribution of pharmacological agents, myokine studies using an *in vitro* exercise model may benefit from a 3D model as it would also better reflect the distribution of contractile forces, and therefore, effects on contraction-mediated myokines. Recent advances in tissue-engineering may soon make this a reliable and feasible option for future research (Khodabukus et al., 2018; Takahashi et al., 2018; Jalal et al., 2021).

Much of the research conducted about AMPK-mediated effects of myokines on metabolism in a pathophysiological context has been in models of type 2 diabetes, and some research has been conducted with obesity models as obesity is a prime risk factor for diabetes (Schnurr et al., 2020). Insulin resistance and subsequent hyperglycemia being hallmarks of diabetes (Reaven, 1988), non-human experimental models replicated this *in vivo via* diet-induced insulin resistance, and *in vitro via* palmitate-induced insulin resistance or high glucose media. IL-6, FGF21, METRNL, and irisin have all been studied in these contexts, with the latter providing the most insight into the beneficial effects of myokine-mediated AMPK signalling. While no specific studies were found to firmly establish a specific animal model as an optimal representative of human myokine signalling, rodents have been found to be viable models of human skeletal muscle physiology in various contexts. Specifically, the C57BL/6 mouse has been reported as a “suitable” model of mitochondrial function in the skeletal muscle, and Sprague-Dawley rats were found to be adequate models of weightlifting and skeletal muscle growth (Wong and Booth, 1988; Jacobs et al., 2013). Garton et al. have also reported various mouse and rat models to provide insight on exercise capacity and health status (Garton et al., 2016). Such models are advantageous as they allow *in vivo* observations, overcoming the greatest downfall of tissue culture models. Given the lack of specific research on inter-species variations in myokine signalling, such differences are difficult to account for. However, the current precedence of mice and rats being reliable models for human skeletal muscle physiology suggests the differences may not be drastic.

Several studies included in this review use recombinant myokine treatments to study their effects on cell signalling. This is an effective method to evaluate causative relationships, as the controlled increase allows for the association of subsequent reactions to the increase in myokine concentration. However, many of these models often utilize supraphysiological concentrations. This poses a challenge to deriving biologically relevant conclusions as it is possible the observed signalling may not take place under physiological conditions. Some studies suggested possible feedback mechanisms or thresholds of saturation where concentrations that were too high may have led to desensitization. Similarly, prolonged exposure to high dosages may lead to receptor downregulation and disruptions to secondary messengers. All of these possibilities may restrict further signalling. If this is in fact the reality, it may cause some studies using only supraphysiological concentrations to observe no reaction. To mitigate these possibilities, it is important to strive to use more physiologically relevant concentrations of myokines and conduct dose-response studies. The absence of data regarding interstitial physiological concentrations of many myokines surrounding the muscle fibers both at rest and following muscle contraction poses an obstacle to accomplishing this. Thus, it is important to pursue investigations to characterize the concentrations of myokines in response to exercise in plasma and more importantly in muscle interstitial fluid to better understand the roles of myokines on skeletal muscle function and metabolism. Furthermore, the study of the effect of individual myokines on AMPK pathway activation and *vice versa* in skeletal muscle may not reflect signalling adaptations *in vivo*, because some myokines are co-regulated during exercise and/or in the context of pathophysiology. As suggested in our previous review (Garneau and Aguer, 2019), research into the relationship between AMPK pathway activation and myokine secretion should account for the potential network interactions between these signalling molecules in muscle.

Numerous studies in this review also use Compound C, also known as dorsomorphin, to inhibit AMPK. While some have described the compound as a “potent, selective, reversible, and ATP-competitive inhibitor of AMPK”, others have found evidence to the contrary (National Center for Biotechnology Information, 2022). Dasgupta and Seibel identified several other kinases which are also inhibited by Compound C, some to comparable levels as AMPK, at concentrations of 10 and 0.1 µM (Dasgupta and Seibel, 2018). Our review includes publications that have used Compound C in concentrations ranging from 10 to 50 µM in the study of ANGPTL4, BDNF, FGF21, METRNL, apelin, irisin, and myostatin. Some of these myokines have been studied in association with some of the kinases identified by Dasgupta et al. (Huang et al., 2007; Whitham et al., 2012; Lee et al., 2015; Luo et al., 2015; Lessard et al., 2018; Xie et al., 2018; Tezze et al., 2019; Qing et al., 2020; Rabiee et al., 2020). The possibility thus exists that the use of Compound C may have confounded some results reviewed in this paper, especially considering the elevated concentrations. However, we are unable to assess this as we did not find studies that directly investigated the effects these kinases may have on myokines in relation to AMPK. Therefore, we suggest that future studies using Compound C in this context are cognisant of this possibility and include supplementary data to verify whether the compound may be skewing their data.

## The AMPK-myokine relationship in physiology and pathophysiology

Several studies reviewed in this paper characterized the interactions between myokines and AMPK in non-pathological models to provide insight into their collective effects, and the nuances of their individual effects, on regulating glucose and lipid metabolism, and in some cases, myogenesis, in response to exercise. It has long been established that within the first few seconds of exercise, glycolysis is the primary source of energy in skeletal muscle until oxidative phosphorylation begins to contribute to ATP production in prolonged exercise (Parolin et al., 1999). Where carbohydrate usage is a function of exercise intensity, β-oxidation continues to be used in longer bouts of exercise (Romijn et al., 1993). The literature reviewed in this paper suggests many myokines facilitate glucose uptake and β-oxidation *via* AMPK in response to exercise. It is thus likely that the myokine-induced AMPK-mediated glucose uptake contributes to glycolysis in exercise by increasing intracellular glucose availability. By facilitating β-oxidation, myokines continue to play a role in energy metabolism as oxidative phosphorylation is used. Together, this may reduce glycogenolysis, increasing glycogen availability and allowing for greater exercise capacity. Furthermore, research has shown that increased β-oxidation may induce a beneficial oxidative phenotype in skeletal muscle (Hénique et al., 2015). Therefore, it may be possible that under physiological conditions, increased β-oxidation facilitated by myokines *via* AMPK in response to exercise may allow humans to capitalize on these benefits. Studies in physiological models are valuable for two reasons. First, they serve to enhance our understanding of the molecular mechanisms of metabolism in exercise physiology which may allow for further studies in leveraging these mechanisms to optimize the metabolic benefits of exercise. Second, and more importantly, they provide us with the understanding of the physiological interactions between myokines and AMPK that can be translated to metabolic adaptations to exercise/muscle contraction. Establishing the baseline allows for the understanding of abnormalities, namely for comparisons between the consequences of physical inactivity (low to null exercise levels) and the benefits of exercise training or a more active lifestyle.

Numerous myokines have been reported to be dysregulated in the context of diabetes (Garneau and Aguer, 2019) and HFD-induced obesity (Chen et al., 2015), and alterations in myokine profile potentially due to physical inactivity has been suggested as a likely contributor to metabolic dysfunction (Eckel, 2019). Many studies reviewed in this paper suggest that myokine-induced AMPK signalling increases insulin sensitivity and glucose uptake to reduce hyperglycemia and counteract the effects of obesity and diabetes. IL-6, FGF21, METRNL, and irisin have all been shown to facilitate this AMPK-mediated process to varying degrees. Given the significant role that exercise plays in the prevention and management of diabetes, it is reasonable to hypothesize that these myokines play a considerable role in maintaining insulin sensitivity and managing blood glucose (Colberg et al., 2010; Kirwan et al., 2017). However, patient adherence to recommendations of exercise has historically been low (Qiu et al., 2012; Kirwan et al., 2017). Although some studies have explored more time-efficient and/or motivating alternative exercise protocols such as high intensity interval training to circumvent this limitation (Chen et al., 2015), this is one of the main reasons for resorting to pharmacological means of managing diabetes. AMPK has long been identified as a druggable target for this purpose, but it has also posed a challenge due to its varied mechanisms of activation and centrality in so many biological processes (Zhang et al., 2009; Hawley et al., 2010). Furthermore, recent research has identified associations between metformin, an anti-hyperglycemia AMPK agonist, and development of sarcopenia (Cetrone et al., 2014; Zhang et al., 2021). This highlights the need to identify better pathways to target AMPK signalling and underscores the importance of understanding myokine-AMPK relationships. Therefore, exploring the molecular interactions between AMPK pathway activation and myokine secretion in the context of the beneficial effects of exercise, specifically in the case of exercise protocols proving as potentially more efficient or appropriate therapies for some patients, could help unveil alternative therapies free of common harmful side-effects. In fact, the importance of this extends beyond diabetes.

Myokines and AMPK have also been implicated in myopathies such as Duchenne Muscular Dystrophy (DMD) (Hunt et al., 2011; Pauly et al., 2012; Zhou et al., 2018; Dong et al., 2021). BDNF, ANGPTL4, FGF21, LIF, and myostatin have all been shown to be dysregulated in DMD, whereas literature suggests AMPK activation may yield positive effects against the disease by contributing to increased muscle function and inducing mitophagy. Upregulated IL-6 signalling, reduced plasma irisin, and impaired AMPK signalling have all been associated with myotonic dystrophy (Dozio et al., 2017; Nakamori et al., 2017; Ravel-Chapuis et al., 2022). Some studies have shown that AMPK activation reduced myotonia, inhibited RNA toxicity, and enabled functional benefits *via* histological improvements, and therefore suggested targeted activation of AMPK signalling as a potential therapeutic approach (Brockhoff et al., 2017; Ravel-Chapuis et al., 2022; Ravel-Chapuis and Jasmin, 2022). As the full molecular mechanism of many of these pathways have yet to be elucidated, understanding myokine-AMPK interactions sheds some light on potential signalling pathways that may be leveraged in these investigations. Similarly, novel studies have implicated both myokines and AMPK to play a role in idiopathic inflammatory myopathies (IIMs). Dysregulation of myokine homeostasis has repeatedly been suggested as a contributor to IIM, placing myokines under the spotlight as potential therapeutic targets. Upregulated IL-6, IL-15, IL-18, and myostatin have been suggested to facilitate autoimmune reactions and muscle weakness (Sugiura et al., 2002; Huang et al., 2015; Bhattarai et al., 2016; Mageriu et al., 2020). On the other hand, recent research has suggested exercise-induced AMPK activation to promote senescence and muscle regeneration to increase muscle strength, as a mitigator of inflammatory myopathy (Saito et al., 2020). Myokines and AMPK have also been shown to be involved in cancer cachexia. IL-6, which is elevated in circulation in cachexia (Iwase et al., 2004), induces adverse downstream effects *via* AMPK signalling in this context (White et al., 2013). Interestingly, AMPK has been found to play a dual role in cachexia depending on the stimulus (Hall et al., 2018; Fix et al., 2021). Despite the effects of aberrant IL-6 regulation, studies have shown exercise to positively regulate AMPK activation and contribute to inhibition of muscle atrophy in cachexia (Tanaka et al., 2019; Fix et al., 2021). Similarly, not all myokines behave the same way in this context. Recent findings have indicated the positive effects of IL-8, IL-15, FGF21, irisin, and myonectin in promoting muscle growth and energy management to mitigate cancer cachexia (Dalamaga, 2013; Manole et al., 2018). Finally, research has also been conducted to investigate positive effects of myokines and AMPK in cognitive (Scisciola et al., 2021) and neurodegenerative diseases (Marinangeli et al., 2016; Lee et al., 2021), though these are beyond the scope of this paper as we focused this review on the autocrine/paracrine effects of myokines and not their endocrine effects.

Thus, the literature clearly demonstrates the value in understanding the interactions between various myokines and AMPK. As the myokines rarely work in isolation *in vivo*, this review illustrates their collective potential in both understanding and managing a plethora of pathologies. Simultaneously, the variations in their individual effects are a testament to the potential that individual myokines may hold for tailored approaches to pathophysiological conditions—emphasizing the importance of thoroughly understanding their roles and signalling interactions.

## Conclusion

**FIGURE 1:**
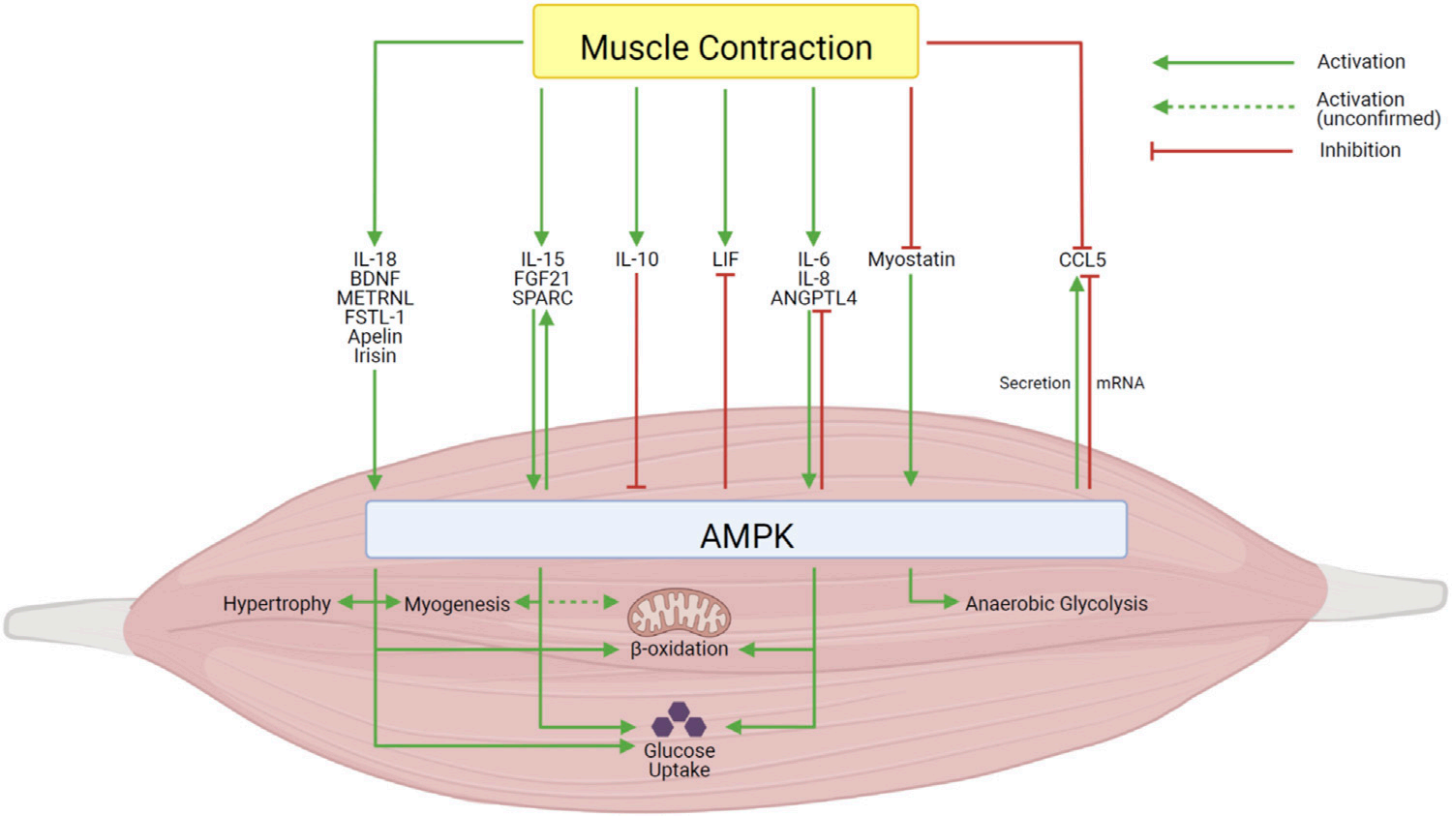
Summary of the effects of the interactions between myokines and AMPK in skeletal muscle. Created with BioRender.com.

This review highlights the relationship between myokines and AMPK as an established mediator of the functional and metabolic effects of exercise on skeletal muscle. Of those myokines, IL-6, IL-8, and ANGPTL4 seem to be involved in a negative feedback loop to maintain energy homeodynamics, though the mechanism of action may not yet be clear. Meanwhile, feedback mechanisms have not been identified for other myokines and further research must be conducted to clarify the regulation of these pathways. A summary of these findings can be found in Table 1 and Figure 1. In this review, we have identified several potential avenues of research to better understand the signalling pathways between myokines and AMPK, and emphasized the need to ensure experimental concentrations are similar to physiological conditions. Elucidating the involvement of myokines in energy metabolism in biologically relevant experimental conditions will provide a holistic understanding of the signalling pathways which activate AMPK during exercise and may also provide insight into possible avenues of investigation in studies of disease and therapy.

**TABLE 1:** Summary of the effects of the interactions between myokines and AMPK in skeletal muscle.

Myokine	Effect of contraction	Effect on AMPK	Metabolic effects	Effect by AMPK	Other effects	Model
IL-6	↑	↑	β-oxidation Glucose uptake	↓ mRNA ↓ secretion		• *Vastus lateralis* of healthy young men MacDonald et al. (2003), Glund et al. (2007), Nylén et al. (2018), healthy fasting humans Wolsk et al. (2010), and humans with diabetes Jiang et al. (2013)
						• Rat EDL, *in vitro* Kelly et al. (2004), Lihn et al. (2008) and *in vivo* Kelly et al. (2009)
						• IL-6 KO mice, *in vivo* Ruderman et al. (2006)
						• Human muscle cells Al-Khalili et al. (2006)
						• L6 myotubes Carey et al. (2006)
						• Palmitate-induced insulin resistant C2C12 cells Tang et al. (2019)
						• Human skeletal muscle cell line derived from the *rectus abdominis* Lihn et al. (2008)
						• Mouse EDL and *soleus* Glund et al. (2009)
						• Whole-body AMPKα2 knockdown, AMPKα1 and AMPKγ3 knockout mice Glund et al. (2009)
						• *Gastrocnemius* of muscle specific AMPKα1α2 double-knockout mice Lantier et al. (2014)
IL-8	↑	↑	Glucose uptake	↓ mRNA		• C2C12 muscle cells Gray and Kamolrat (2011)
						• Human skeletal muscle cell line derived from the *rectus abdominis* Lihn et al. (2008)
						• Rat EDL, *in vitro* Lihn et al. (2008)
IL-10	↑	↓				• *Tibialis anterior* of homozygous IL-10 deficient mice Wang et al. (2014)
						• Rat skeletal muscle Park et al. (2017)
IL-15	↑	↑	β-oxidation* Glucose uptake	↑ mRNA		• L6 myotubes Nadeau et al. (2019)
						• EDL or *gastrocnemius* of IL-15 overexpressing mice Fujimoto et al. (2019)
						• C2C12 muscle cells Gray and Kamolrat (2011), Krolopp et al. (2016)
						• AMPKα2 dominant negative mice Abbott et al. (2012)
						• Muscle-specific AMPKβ1/2 deficient mice Crane et al. (2015)
IL-18	↑	↑	β-oxidation			• L6 muscle cells Lindegaard et al. (2013)
						• Isolated mouse *soleus* muscle strips Lindegaard et al. (2013)
						• Mouse *tibialis anterior* made to overexpress IL-18 *via* IL-18cDNA-electroporation Lindegaard et al. (2013)
ANGPTL4	↑	↑	β-oxidation	↓ mRNA ↓ protein		• C2C12 myotubes Catoire et al., (2014), Chang et al. (2018)
						• Human primary myotubes Catoire et al., (2014)
						• *Soleus* and *gastrocnemius* of exercised mice Chang et al. (2018)
						• Exercising and non-exercising ANGPTL4 KO mice Chang et al. (2018)
BDNF	↑	↑	β-oxidation			• Human skeletal muscle, *in vivo* Matthews et al. (2009)
						• C2C12 muscle cells Matthews et al. (2009), Yang et al. (2019)
						• L6 myotubes Matthews et al. (2009)
						• Rat EDL, *ex vivo* Matthews et al. (2009)
						• *Gastrocnemius* of muscle-specific BDNF KO mice Yang et al. (2019)
CCL5	↓			↑ mRNA ↓ secretion		• C2C12 myotubes Ishiuchi et al. (2018)
FGF21	↑	↑	Glucose uptake	↑ mRNA	Myogenesis	• C2C12 with RNAi-mediate KD of FGF21 Liu et al. (2017)
						• Whole body FGF21 KO and HFD-induce obese mice Kim et al. (2019)
						• L6 myotubes Zhou et al. (2016), Nylén et al. (2018)
						• Muscle-specific Cpt1b^−/−^ mice Vandanmagsar et al. (2016)
						• Healthy lean humans Vandanmagsar et al. (2016)
FSTL-1	↑	↑	Glucose uptake			• Primary human skeletal muscle Görgens et al. (2013)
						• Primary rodent myotubes Lee et al. (2017)
						• L6 myotubes Lee et al. (2017)
LIF	↑			↓ mRNA		• Human primary skeletal muscle cells Nylén et al. (2018)
METRNL	↑	↑	Glucose uptake			• C2C12 myotubes Jung et al. (2018), Lee et al. (2020)
						• *Quadriceps femoris* of chronic exercised mice Lee et al. (2020)
						• EDL of healthy, obese, and diabetic mice, *in vivo* Lee et al. (2020)
						• Muscle-specific AMPK β1β2 KO mice Lee et al. (2020)
SPARC	↑	↑	Glucose uptake	↑ mRNA ↑ protein		• COS-7 primate kidney cells overexpressing flag-tagged SPARC and myc-tagged AMPKα1 Song et al. (2010)
						• HepG2 human hepatic cells Song et al. (2010)
						• L6 myotubes and myoblasts Song et al. (2010)
						• Whole-body SPARC-knockout mice Aoi et al. (2019)
						• AMPKγ3-knockout mice Aoi et al. (2019)
						• C2C12 myotubes Aoi et al. (2019)
Apelin	↑	↑	Glucose uptake		Myogenesis Hypertrophy	• *Soleus* of eNOS-deficient mice, *ex vivo* Dray et al. (2008)
						• DN AMPK mice Dray et al. (2008), Vinel et al. (2018)
						• Myotubes from young and aged humans Vinel et al. (2018)
Irisin	↑	↑	β-oxidation Glucose uptake			• L6 cells Lee et al. (2015)
						• Irisin overexpressing C2C12 muscle cells Yano et al. (2020) and diet-induced insulin resistant C2C12 myotubes, *in vitro* Xin et al. (2016)
						• *Gastrocnemius* of diet-induced type 2 diabetic mice *in vivo* Xin et al. (2016)
						• *Soleus* muscle from irisin-treated mice, *in vivo* Yano et al. (2020)
Myonectin (CTRP15)	↑					• *Soleus* and plantaris of mice Seldin et al. (2012)
						• C2C12 myotubes Chen et al. (2010), Seldin et al. (2012)
Myostatin	↓	↑	Anaerobic glycolysis			• Mouse EDL Zhang et al. (2011)

↑ indicates increase, ↓ indicates decrease, and * indicates unconfirmed effect.
